# Computerized Self-Reported Medical History Taking to Support Early Rule Out of Major Adverse Cardiac Events in Patients With Acute Chest Pain: Post Hoc Analysis of the CLEOS-CPDS Prospective Cohort Study

**DOI:** 10.2196/76087

**Published:** 2026-02-11

**Authors:** Helge Brandberg, Carl Johan Sundberg, Jonas Spaak, Sabine Koch, Thomas Kahan

**Affiliations:** 1 Division of Cardiovascular Medicine Department of Clinical Sciences, Danderyd Hospital Karolinska Institutet Stockholm Sweden; 2 Department of Learning, Informatics, Management and Ethics Karolinska Institutet Stockholm Sweden; 3 Department of Physiology & Pharmacology Karolinska Institutet Stockholm Sweden

**Keywords:** chest pain, risk assessment, coronary artery disease, emergency service, hospital, medical informatics, artificial intelligence, medical history taking

## Abstract

**Background:**

Self-reported, computerized history taking (CHT) may enable efficient collection of medical histories for acute chest pain management.

**Objective:**

The primary aim is to determine the diagnostic performance of 4 CHT-derived chest pain risk scores for ruling out 30-day major adverse cardiac events (MACEs) or acute coronary syndrome (ACS). The secondary aim is to assess their impact on patient disposition in the emergency department (ED).

**Methods:**

This is a prospective cohort study conducted at a tertiary hospital ED in Stockholm, Sweden. Clinically stable adults (≥18 years) with chest pain and an electrocardiogram (ECG) not indicating an acute disease requiring immediate care provided medical histories via a tablet-based CHT program (Clinical Expert Operating System [CLEOS]). CHT data and ECG interpretations and troponin values were used to calculate the History, ECG, Age, Risk Factors, and Troponin (HEART) score, Danderyd HEART (D-HEART) score, Emergency Department Assessment of Chest Pain Score combined with an Accelerated Diagnostic Protocol (EDACS-ADP), and Troponin-only Manchester Acute Coronary Syndrome (T-MACS). The primary outcome was 30-day ACS; the secondary outcome was 30-day MACE (ACS, revascularization, or cardiovascular death).

**Results:**

Among 1000 participants (age: mean 55 years, SD 17 years; 456/1000, 45.60%, women), risk scores could be calculated in 838 (83.80%). Within 30 days, 65 (6.50%) participants experienced ACS, and 72 (7.20%) had a MACE. Negative predictive values were 0.99 (95% CI 0.97-1.00) for both outcomes. Sensitivity for MACE was 0.91 (95% CI 0.81-0.97) for HEART, 0.94 (95% CI 0.86-0.98) for D-HEART, 0.94 (95% CI 0.86-0.98) for EDACS-ADP, and 0.97 (95% CI 0.90-1.00) for T-MACS, with similar results for ACS. As many as 89 of the 528 (16.9%) patients admitted could be reclassified from “nonlow risk” to “low risk.” Among reclassified patients, 30-day MACE or ACS occurred in 0-4 cases; miss rates were below 1% for D-HEART (4/416, 0.96%) and T-MACS (2/286, 0.7%), but exceeded 1% for HEART (6/406, 1.5%) and EDACS-ADP (4/346, 1.2%).

**Conclusions:**

Automated, self-reported CHT provided sufficient data to calculate 4 chest pain risk scores in 838 of 1000 (83.80%) patients with acute chest pain, with score calculation dependent on physician-interpreted ECGs. These CHT-derived risk scores demonstrated good diagnostic performance for ruling out 30-day MACE and ACS. Performance was broadly comparable with prior studies using physician-acquired scores, although suggested safety thresholds were primarily met by D-HEART and T-MACS. The improved safety of D-HEART compared with HEART is likely attributable to the incorporation of serial 0/1-hour troponin testing. Use of CHT-derived risk scores may reclassify a substantial fraction of admitted patients as “low risk,” potentially supporting discharge decisions in selected patients, while admission may still be required for non-ACS reasons. However, any gains in discharge rates should be weighed against the possibility of missed events among reclassified patients. Multicenter studies are needed to confirm generalizability, operational feasibility, and safety.

**Trial Registration:**

ClinicalTrials.gov NCT03439449; https://clinicaltrials.gov/ct2/show/NCT03439449

**International Registered Report Identifier (IRRID):**

RR2-10.1136/bmjopen-2019-031871

## Introduction

Chest pain, a common chief complaint in emergency departments (EDs) worldwide [1,2], contributes to overcrowding in these settings [3]. Prompt and accurate management is crucial, not only for identifying life-threatening conditions such as acute coronary syndrome (ACS; acute myocardial infarction or unstable angina pectoris), but also for the safe discharge of the majority of patients with benign conditions. An integrated assessment combining medical history, signs and symptoms, electrocardiogram (ECG), and high-sensitivity cardiac troponins (hs-cTn) is recommended for initial short-term risk stratification [4]. To consolidate these observations, risk scores are recommended for structured, individualized patient assessment [5,6]. Their clinical utility is supported by substantial evidence [7-9], and recent data have shown that risk score implementation can reduce the risk of major adverse cardiac events (MACEs) by half among patients with chest pain discharged from the ED [10]. However, chest pain risk scores remain underutilized in EDs [11], and when used, issues such as miscalculation [12] or discordance among physicians [13] are common.

Digital tools are increasingly integrated into health care, offering potential enhancements in efficiency and decision-making [14,15]. In acute chest pain management, international societies advocate improved symptom classification methods using machine learning [16]. In addition to biometric data such as vital signs or ECG, prompt and reliable acquisition of medical history data is essential for the use of these techniques. Computerized history taking (CHT) is a possible solution, offering an automated, structured, and standardized approach to self-reported medical history. Previous studies indicate that CHT can assist physicians by providing a more standardized, detailed, and complete medical history in both emergent and nonemergent settings [17-20]. Studies have also shown that CHT is well received by patients with acute chest pain, with a majority able to interact effectively and provide sufficient data for risk stratification [21-23]. However, there is a knowledge gap regarding the impact of CHT on clinical management and outcomes in acute care settings [24]. This study proposes a standardized, automated approach to collect medical history data, enabling real-time use of clinical decision-support tools. This approach has the potential to significantly impact the management of the large population of patients presenting to EDs with chest pain.

We hypothesized that risk scores populated with CHT data would reliably identify patients with acute chest pain at low risk for MACE or ACS, enabling early rule out and thereby reducing unnecessary admissions and diagnostic testing without compromising patient safety. Our aims were therefore, first, to determine the overall diagnostic performance of 4 chest pain risk scores populated with CHT data for 30-day MACE (defined as ACS, coronary artery revascularization, or cardiovascular death) or ACS rule out; and second, to assess the potential impact of this approach on patient disposition among those presenting to the ED with acute chest pain.

## Methods

### Study Design and Setting

The Clinical Expert Operating System Chest Pain Danderyd Study (CLEOS-CPDS; ClinicalTrials.gov identifier: NCT03439449) was a prospective cohort study designed to determine the value of self-reported CHT in acute chest pain management [25]. This study reports diagnostic accuracy and adheres to the STARD (Standards for Reporting Diagnostic Accuracy) 2015 guideline [26].

### Participants

Patients presenting to Danderyd University Hospital, a tertiary hospital in Stockholm, Sweden, were consecutively enrolled between October 1, 2017, and May 16, 2019. Eligible participants were women and men aged 18 years or older, fluent in Swedish, with a presenting complaint of chest pain recorded by an ED triage nurse or registrar; a nondiagnostic initial ECG, serum markers, or both, for an acute disease requiring immediate care; and clinical stability, as defined by the Rapid Emergency Triage and Treatment System (RETTS) levels orange, yellow, green, and blue [27]. Exclusion criteria were inability to complete a CHT interview (eg, agitation, severely impaired vision, or confusion). Among patients considered eligible but not enrolled, the most common reasons were language barriers (eg, inability to read Swedish), patients feeling too tired, and difficulties using a tablet [22]. All patients included in the study were initially managed as suspected ACS according to standard care protocols recommended by regional guidelines [28]. Data from the CHT and the calculated risk scores were not accessible to the treating physicians or other health care professionals and were used solely for clinical research purposes.

### Interventions

#### Computerized History Taking

Medical history was collected using the CHT software CLEOS on tablets (iPad; Apple Inc), as described in detail previously [25,29]. Briefly, CLEOS is an expert system, a form of artificial intelligence software, that uses a rule-based approach to guide the history-taking process. It incorporates a comprehensive medical knowledge base, algorithmically represented by decision trees comprising over 17,000 decision nodes. By directly interacting with the patient, CLEOS automates history taking through a series of structured questions, mainly in text format (ie, yes/no or multiple-choice questions), as well as image-based questions. A limited number of free-text entries are available for instances in which patients are unable to select a suitable option, such as when describing the quality of their pain. Based on prior responses and their clinical relevance, CLEOS adapts the interview and dynamically determines the next most appropriate questions, emulating clinical reasoning as data are continuously collected [29]. In a study evaluating interrater reliability between data collected using CHT and physician-acquired data, we observed high agreement for traditional risk factors (eg, diabetes mellitus or hypercholesterolemia), but low to moderate agreement for chest pain characteristics (eg, pain radiating to the arm or relief by rest) [30]. As previously reported, the median time required to collect sufficient information to calculate the HEART score was 23 (IQR 18-31) minutes [22].

#### Chest Pain Risk Scores

The History, ECG, Age, Risk Factors, and Troponin (HEART) score; Emergency Department Assessment of Chest Pain Score combined with an Accelerated Diagnostic Protocol (EDACS-ADP); and Troponin-only Manchester Acute Coronary Syndrome (T-MACS) score were calculated as described in their respective derivation studies [31-33]. When assessing the History component of the HEART score in this retrospective analysis, the traditional clinical classification of suspected anginal symptoms was used: (1) central chest pain, (2) triggered by physical or emotional exertion, and (3) relieved by rest or nitrates. Based on the number of criteria met, patient history was categorized as highly (3 criteria met), moderately (2 criteria met), or slightly suspicious (none or 1 criterion met). This predefined, symptom-based approach to probability assessment, used as an aid in the clinical diagnosis of coronary artery disease (Diamond-Forrester prediction rule) [34], reflects commonly applied principles for reconstructing the HEART History component in several validation studies [35,36]. However, this approach differs from the derivation study and subsequent validation study by the same authors, in which clinician judgment, rather than a predefined set of criteria, was used to evaluate the likelihood of chest pain being related to MACE [33,37]. The Danderyd HEART (D-HEART) score was calculated according to guidelines in Region Stockholm [28], combining the HEART score with hs-cTn assays using the European Society of Cardiology (ESC) 0/1-hour rule-out and rule-in algorithm [38,39]. By contrast, the HEART score in this study was calculated using a single baseline hs-cTn measurement, in accordance with the derivation study. We defined a clinically decisive risk score as “nonlow risk,” indicating the need for hospitalization, if D-HEART ≥4, HEART ≥4, EDACS ≥16, T-MACS ≥0.02, or if troponin met rule-in criteria using the ESC 0/1-hour algorithm [4]. For T-MACS, we evaluated both the <0.02 (very low risk) and <0.05 (low risk) thresholds, as they represent distinct clinical pathways (immediate discharge vs clinical observation with further serial troponin testing) and were defined in the derivation study [32]. Detailed descriptions of chest pain characteristics, risk factor components, and interpretation (ie, “nonlow risk” thresholds) for D-HEART, HEART, EDACS, and T-MACS are provided in Table 1.

**Table 1:** Components and interpretation of assessed risk scores.

Component		Risk score				
		D-HEART^a^	HEART^b^	EDACS-ADP^c^	T-MACS^d^	
Age		✓	✓	✓	N/A^e^	
Sex		N/A	N/A	✓	N/A	
**Chest pain characteristics**						
	Central chest pain	✓	✓	N/A	N/A	
	Provoked by physical exertion or emotional stress	✓	✓	N/A	N/A	
	Relieved by rest or nitrates	✓	✓	N/A	N/A	
	Diaphoresis	N/A	N/A	✓	✓	
	Radiation to arm or shoulder	N/A	N/A	✓	N/A	
	Radiation to right arm or shoulder	N/A	N/A	N/A	✓	
	Pain occurred/worsened with inspiration	N/A	N/A	✓	N/A	
	Pain is reproduced by palpation	N/A	N/A	✓	N/A	
	Worsening or crescendo angina	N/A	N/A	N/A	✓	
	Pain associated with vomiting	N/A	N/A	N/A	✓	
**Risk factors**						
	Atherosclerotic disease	✓	✓	N/A	N/A	
	Known coronary artery disease (not stroke)	N/A	N/A	✓	N/A	
	Diabetes mellitus	✓	✓	✓	N/A	
	Current smoker	✓	✓	✓	N/A	
	Family history of premature coronary artery disease	✓	✓	✓	N/A	
	Hypertension	✓	✓	✓	N/A	
	Hypercholesterolemia	✓	✓	✓	N/A	
	Reported obesity	✓	✓	N/A	N/A	
**Electrocardiogram, vital signs, and biomarkers**						
	Signs of ischemia on electrocardiogram	✓	✓	✓	✓	
	Hypotension, systolic blood pressure <100 mmHg	N/A	N/A	N/A	✓	
	Elevated biomarker (troponin)	✓	✓	✓	✓	
Possible range of score		0 to 8	0 to 10	–10 to 34	0 to 1	
Threshold for “nonlow risk”		≥4^f^	≥4	≥16^g^	≥0.02	

^a^D-HEART: Danderyd HEART.

^b^HEART: History, ECG, Age, Risk Factors, and Troponin.

^c^EDACS-ADP: Emergency Department Assessment of Chest Pain Score combined with an Accelerated Diagnostic Protocol.

^d^T-MACS: Troponin-only Manchester Acute Coronary Syndrome.

^e^N/A: not applicable.

^f^And a rule-in troponin result using the European Society of Cardiology 0/1-hour algorithm [38].

^g^And new ischemia on electrocardiogram and/or a rule-in 0- or 2-hour troponin result.

### Data Collection

At presentation to the ED, all patients with acute chest pain were triaged to determine urgency either by a cardiology consultant or a senior cardiology resident (8 AM to 5 PM) or by a triage nurse using the RETTS protocol (5 PM to 8 AM) [27]. After ECG and biomarker acquisition during triage, patients underwent a more thorough examination and standard history taking by the attending physician, either in the ED cardiology unit—operating 24 hours a day and staffed by cardiology consultants or senior residents with limited access to noninvasive cardiac imaging techniques—or in the inpatient day-care unit. The day-care unit, operational from 8 AM to 5 PM, functions as an observational ward, is staffed by cardiology consultants, and provides ready access to both invasive and noninvasive cardiac imaging techniques. Details regarding patient disposition have been reported in previous publications [22,25].

The participant enrollment process has been described in detail previously [22,25]. Briefly, patients were invited to participate in the study by a research staff member in the ED cardiology unit or the inpatient day-care unit. Patients were provided with standardized information and given the opportunity to ask questions before signing a consent form to provide informed consent. CHT was conducted during waiting times in the ED, either before or after the initial physician consultation. The CHT interview could be paused during standard clinical procedures (eg, blood sampling or radiographic examinations) and resumed afterward. Discontinuation of CHT occurred upon completion, at the patient’s request, or upon discharge home or admission to a ward. The CHT interview did not interfere with standard management, and ED staff had no access to data collected through CHT.

Data relevant to this study were extracted from the electronic health record (EHR; TakeCare; CompuGroup Medical Sweden AB) by research staff and from the CHT database. To align with variables used in the risk scores, we predefined variables to be collected and established an interpretation scheme that allowed all variables to be converted into a binary format (yes/no responses; see an example of the interpretation scheme for crescendo angina pectoris in [30]). Demographic information (age and sex) for the general ED population at Danderyd University Hospital during the study period was extracted from the EHR system using QlikView version 12.10 (QlikTech International AB).

ECG findings, interpreted by both physicians and computer algorithms, were collected by a research assistant from the EHR. To specifically evaluate the independent contribution of CHT to chest pain management, physician-reported ECG interpretations were deliberately used to populate the risk scores. In cases in which a physician report was missing (n=58), the automated ECG interpretation (EC Sense ECG; Cardiolex Medical AB) was imputed. A detailed description of ECG findings and their categorization is provided in Table S1 in Multimedia Appendix 1. Blood samples were collected according to standard procedures at the Karolinska University Laboratory, Stockholm. The detection limit of the assay was 5 ng/l, with a 99th percentile upper reference limit—used to identify elevated levels indicative of myocardial injury—of 14 ng/l [40].

All variables required for each risk score were extracted from the EHR and CHT and transformed according to the predefined interpretation scheme. CHT responses were manually mapped post hoc, and risk scores were calculated retrospectively.

### Outcome Measures

The primary outcome was defined as a diagnosis of ACS [25]. To facilitate comparison with other diagnostic accuracy studies of acute chest pain, we also report 30-day MACE as a secondary outcome, comprising an ACS diagnosis, coronary artery revascularization, or cardiovascular death within 30 days. According to the study protocol, the diagnosis of ACS was verified by a board-certified cardiologist in accordance with prevailing European guidelines at the time of the study [41,42]. The cardiologist then assigned the corresponding International Statistical Classification of Diseases and Related Health Problems, 10th Revision (ICD-10) codes: I20.0 (unstable angina pectoris), I21.0-9 (acute myocardial infarction), or I24.0-9 (other acute ischemic heart diseases) [43,44]. Previous studies have shown that the overall positive predictive value (PPV) of Swedish discharge ICD codes is 85%-95% for most diagnoses, including acute myocardial infarction [45,46]. Data on death and revascularization were retrieved through review of the regional EHR system, which is shared by all hospitals and cardiology outpatient clinics in Region Stockholm. The regional EHR system is linked to the national population registry, providing complete information on mortality status. No active follow-up or separate linkage to a national registry was performed. Further details are available in the study protocol [25].

Our previous work shows that CHT provides more complete information on key medical history variables than that reported by physicians in the EHR [30]. Consequently, sufficient information for risk score calculation was available for fewer than 30% of patients when using EHR data alone, compared with approximately three-quarters when using CHT data [30]. As a result, reliable retrospective calculation of a complete physician-derived HEART score was not possible, making a direct head-to-head comparison with the CHT-derived score infeasible. To evaluate how CHT might influence clinical management, we therefore used patient disposition as a proxy for the attending physician’s overall risk assessment. Participants discharged from the ED to home were classified as “low risk,” whereas those admitted to a ward or day-care unit were categorized as “nonlow risk.”

### Statistical Analysis

In this post hoc analysis evaluating diagnostic accuracy, a risk score populated with CHT data was considered the index test, and the occurrence of MACE or ACS was considered the reference test. Descriptive statistics are presented as means (SDs), 95% CIs, or proportions, as appropriate. Differences between groups with and without MACE or ACS were assessed using the Student *t* test for continuous variables and the Pearson chi-square test for binomial and categorical variables. Diagnostic accuracy was evaluated using cross-tabulation of the index test against the reference test. For each index test, performance was assessed by calculating sensitivity, specificity, PPV, and negative predictive value (NPV) for the reference test. The Pearson chi-square test was used to compare NPVs between index tests. Receiver operating characteristic curves were generated for each risk score, and the Hanley and McNeil method [47] was used to test for differences between areas under the receiver operating characteristic curve (AUCs). The miss rate was defined as the proportion of missed cases in the rule-out group. All analyses were performed using STATA, version 14.2 (StataCorp).

The sample size was determined based on the targeted precision of sensitivity and specificity in the original study objective, as previously described [25]. Briefly, because the prevalence of MACE and ACS in the study population was unknown, the calculation was based on an assumed prevalence of 0.5 (50%), which maximizes the estimated sample size. To achieve a 0.03 (3%) precision for sensitivity and specificity, as calculated using nQuery, version 7.0 (Statistical Solutions Ltd), 1000 participants were required.

### Ethical Considerations

This study complied with the Declaration of Helsinki and was approved by the Stockholm Regional Ethical Committee (now the Swedish Ethical Review Authority; reference number 2015/1955-31). All participants received oral and written information about the study and provided written informed consent before inclusion. Study data were deidentified before analysis, and only coded study IDs were used in the research dataset. Participants did not receive any financial or other compensation for their participation.

## Results

### Study Population Characteristics

**Figure 1:**
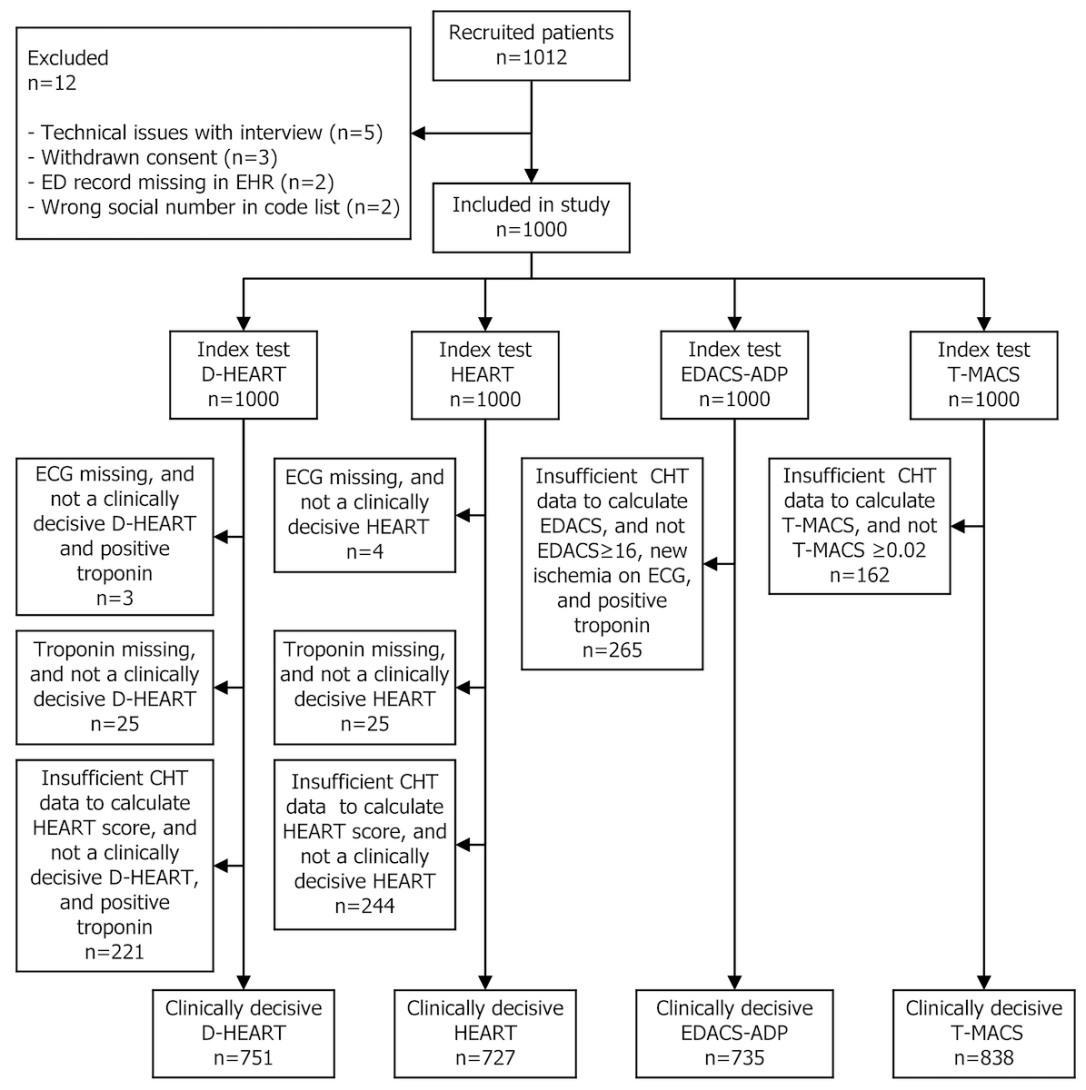
Study flow diagram. CHT: computerized history taking; D-HEART: Danderyd HEART; ECG: electrocardiogram; ED: emergency department; EDACS-ADP: Emergency Department Assessment of Chest Pain Score combined with an Accelerated Diagnostic Protocol; EHR: electronic health record; HEART: History, ECG, Age, Risk Factors, and Troponin; T-MACS: Troponin-only Manchester Acute Coronary Syndrome.

During the study period, a total of 13,044 patients presented to the ED with a chief complaint of chest pain. During periods with on-duty research staff (office hours, evenings, and weekends), 1000 patients were consecutively included (Figure 1). The age and sex distribution of the study population (age: mean 55.3 years, SD 17.4 years; 456/1000, 45.60% female) closely aligned with that of the general chest pain population at Danderyd University Hospital (age: mean 57.6 years, SD 19.1 years; 49% female). Further baseline characteristics collected by CHT and selected ED variables, as well as patient disposition, are summarized in Tables 2 and 3.

**Table 2:** Demography and background of all included patients divided into groups depending on outcome (30-day MACE^a^ and ACS^b^).^c^

Characteristics			All					MACE					Non-MACE					*P* value			ACS					Non-ACS					*P* value
			Value		n		Value			n		Value			n					Value			n		Value			n			
Age (years), mean (SD)			54.7 (17.2)		1000		67.6 (11.0)			72		53.7 (17.1)			928		<.001			68.0 (10.9)			64		53.8 (17.1)			936		<.001	
Sex (females), n (%)			456 (45.60)		1000		19 (26.39)			72		437 (47.09)			928		.001			18 (28.13)			64		438 (46.79)			936		.004	
BMI (kg/m^2^), mean (SD)			26.4 (4.7)		1000		26.6 (4.3)			72		26.3 (4.7)			928		.63			26.2 (3.9)			64		26.4 (4.7)			936		.82	
Diabetes mellitus type 1 or 2, n (%)			62 (7.87)		788		8 (15.69)			51		54 (7.33)			737		.03			5 (11.36)			44		57 (7.66)			744		.37	
Ongoing lipid-lowering medication, n (%)			128 (20.00)		640		21 (47.73)			44		107 (17.95)			596		<.001			18 (46.15)			39		110 (18.30)			601		<.001	
Hypertension, n (%)			316 (41.31)		765		34 (64.15)			53		282 (39.61)			712		<.001			31 (67.39)			46		285 (39.64)			719		<.001	
Family history of coronary artery disease, n (%)			200 (25.84)		774		18 (37.50)			48		182 (25.07)			726		.06			17 (41.46)			41		183 (24.97)			733		.02	
**Known coronary artery disease, n (%)**			137 (16.10)		851		31 (55.36)			56		106 (13.33)			795		<.001			27 (55.10)			49		110 (13.72)			802		<.001	
	History of angina pectoris	88 (10.35)		850		23 (41.07)			56		65 (8.19)			794		<.001			20 (40.82)			49		68 (8.49)			801		<.001		
	History of myocardial infarction	82 (9.65)		850		21 (37.5)			56		61 (7.68)			794		<.001			18 (36.73)			49		64 (7.99)			801		<.001		
	History of percutaneous coronary intervention	76 (9.12)		833		16 (29.09)			55		60 (7.61)			778		<.001			13 (27.08)			48		63 (8.03)			785		<.001		
	History of coronary artery bypass graft	18 (2.16)		833		10 (18.18)			55		8 (1.03)			778		<.001			9 (18.75)			48		9 (1.15)			785		<.001		
Current smoker, n (%)			90 (11.39)		790		5 (9.43)			53		85 (11.53)			737		.64			5 (10.87)			46		84 (11.29)			744		.91	
**Region of birth, n (%)**																															
	Nordic countries	829 (82.90)		1000		63 (87.50)			72		766 (82.54)			928		.28			55 (85.94)			64		769 (82.16)			936		.50		
	Europe (outside the Nordic countries)	46 (4.60)		1000		1 (1.39)			72		45 (4.85)			928		.18			1 (1.56)			64		45 (4.81)			936		.23		
	Outside Europe	125 (12.50)		1000		8 (11.11)			72		117 (12.61)			928		.71			8 (12.50)			64		116 (12.39)			936		>.99		
**Occupational status, n (%)**																															
	Active worker (employed or student)	616 (61.60)		1000		26 (36.11)			72		590 (63.58)			928		<.001			23 (35.94)			64		589 (62.93)			936		<.001		
	Not at work (unemployed or on sick leave)	69 (6.90)		1000		1 (1.4)			72		68 (7.33)			928		.06			1 (1.56)			64		67 (7.16)			936		.08		
	Retired	315 (31.50)		1000		45 (62.50)			72		270 (29.09)			928		<.001			40 (62.50)			64		274 (29.27)			936		<.001		
Arrived at emergency department by ambulance, n (%)			189 (20.61)		917		14 (24.14)			58		175 (20.37)			859		.49			13 (25.49)			51		176 (20.32)			866		.37	
Ongoing chest pain during computerized history taking, n (%)			544 (60.99)		892		19 (34.55)			55		525 (62.72)			837		<.001			16 (33.33)			48		524 (62.09)			844		<.001	

^a^MACE: major adverse cardiac event.

^b^ACS: acute coronary syndrome.

^c^Self-reported medical history data derived from computerized history taking.

**Table 3:** Vital signs, electrocardiogram, circulating biomarkers, and disposition of all included patients divided into groups depending on outcome (30-day MACE^a^ and ACS^b^) or not.^c^

Characteristic				All				MACE					Non-MACE					*P* value			ACS					Non-ACS					*P* value
				Value		n		Value		n		Value			n					Value			n		Value			n			
**Vital parameters at triage, mean (SD)**																															
	Systolic blood pressure (mmHg)		143 (22)		995		148 (22)		72		143 (22)			923		.06			148 (22)			64		143 (21)			931		.08		
	Diastolic blood pressure (mmHg)		83 (13)		993		81 (14)		72		84 (13)			921		.18			82 (15)			64		83 (13)			929		.30		
	Heart rate (beats/minute)		77 (16)		984		74 (15)		72		77 (16)			912		.15			74 (16)			64		77 (16)			920		.19		
	Respiration rate (breaths/minute)		16 (3)		984		16 (2)		72		16 (3)			912		.85			16 (2)			64		16 (3)			920		.86		
	Body temperature (°C)		36.8 (0.4)		947		36.6 (0.4)		70		36.8 (0.4)			877		<.001			36.6 (0.4)			63		36.8 (0.4)			884		.001		
**Electrocardiogram, n (%)^d^**																															
	New signs diagnostic for ischemia		68 (6.8)		994		14 (19.7)		71		54 (5.9)			923		<.001			14 (22.2)			63		53 (5.7)			931		<.001		
	New nonspecific ST-T changes		131 (13.2)		994		20 (28.2)		71		111 (12.0)			923		<.001			16 (25.4)			63		115 (12.4)			931		.003		
	Normal or known ST-T alterations		795 (80.0)		994		37 (52.1)		71		758 (82.1)			923		<.001			33 (52.4)			63		758 (81.4)			931		<.001		
**High-sensitive troponin T values, n (%)**																															
	>3× normal limit (>42 ng/l)		47 (4.8)		970		29 (40.8)		71		18 (2.0)			899		<.001			28 (44.4)			63		19 (2.1)			907		<.001		
	1-3× normal limit (15-42 ng/l)		112 (11.5)		970		19 (26.8)		71		93 (10.3)			899		<.001			16 (25.4)			63		96 (10.6)			907		<.001		
	Normal limit^e^ (≤14 ng/l)		811 (83.6)		970		23 (32.4)		71		788 (87.7)			899		<.001			19 (30.2)			63		786 (86.7)			907		<.001		
	**5-14 ng/l**		397 (40.9)		970		15 (21.1)		71		367 (40.8)			899		.001			11 (17.5)			63		368 (40.6)			907		.004		
		1-hour troponin T elevated (>2 ng/l)	21 (2.2)		970		2 (2.8)		71		19 (2.1)			899		.69			2 (3.2)			63		19 (2.1)			907		.57		
	<5 ng/l		414 (42.7)		970		4 (5.6)		71		410 (45.6)			899		<.001			4 (6.3)			63		407 (44.9)			907		<.001		
**Admitted to the ward or day-care unit, n (%)**				528 (53.3)		990		69 (95.8)		72		459 (50.0)			918		<.001			62 (96.9)			64		466 (50.3)			926		<.001	
	Ward (not via day-care unit)		203 (20.5)		990		61 (84.7)		72		141 (15.4)			918		<.001			57 (89.1)			64		146 (15.8)			926		<.001		
	**Day-care unit**		325 (32.8)		990		7 (9.7)		72		318 (34.6)			918		<.001			5 (7.8)			64		320 (34.6)			926		<.001		
		Day-care unit then to ward	33 (3.3)		7 (9.7)		7 (10)		72		26 (2.8)			918		.002			5 (7.8)			64		28 (3.0)			926		.04		
		Day-care unit then sent home	292 (29.5)		990		0 (0)		72		292 (31.8)			918		<.001			0 (0)			64		292 (31.5)			926		<.001		

^a^MACE: major adverse cardiac event.

^b^ACS: acute coronary syndrome.

^c^Data were derived from the electronic health record.

^d^ For definitions of electrocardiogram interpretation, see Table S1 in Multimedia Appendix 1).

^e^99th percentile (for definitions of electrocardiogram interpretation, see Table S1 in Multimedia Appendix 1).

### Endpoints Reached Within 30 Days

Among the 1000 included patients, 72 experienced a MACE, including 65 with an ACS; 7 underwent coronary revascularization without an ACS diagnosis. No cases of cardiovascular death occurred (1 noncardiac death due to advanced lung cancer was excluded). Three patients diagnosed with unstable angina pectoris were not revascularized. The types of MACE and ACS are presented in Table 4. MACE and ACS were associated with increasing age, male sex, diabetes mellitus, lipid-lowering therapy, hypertension, and a history of coronary artery disease (Table 2). Primary diagnoses for participants discharged from either the ED or the day-care unit, as well as those hospitalized, are reported in Tables 5 and 6, respectively. Three patients were diagnosed with pulmonary embolism at the ED visit, assessed as low risk, and discharged according to local clinical protocols with appropriate anticoagulant therapy and dedicated specialist outpatient follow-up; these cases therefore do not represent missed or chronic diagnoses. During the study period, 2 patients receiving standard care experienced a MACE within 30 days after ED discharge to home.

**Table 4:** Major adverse cardiac events within 30 days among the 1000 participants.^a^

Outcome							7 days^b^, n			30 days^c^, n			Total, n
**Major adverse cardiac event**							70			2			72
	**Acute coronary syndrome**						64			1			65
		**Unstable angina pectoris (I20.0)**					22			0			22
			**Revascularization**			19			0			19	
				Percutaneous coronary intervention	16			0			16		
				Coronary artery bypass grafting	3			0			3		
			No revascularization			3			0			3	
		**Acute subendocardial myocardial infarction (I21.4)**					33			1			34
			**Revascularization**			24			1			25	
				Percutaneous coronary intervention	23			1			24		
				Coronary artery bypass grafting	1			0			1		
			No revascularization			9			0			9	
		**Acute myocardial infarction, unspecified, type 1 (I21.9 + I98.1)**					6			0			6
			**Revascularization**			4			0			4	
				Percutaneous coronary intervention	4			0			4		
				Coronary artery bypass grafting	0			0			0		
			No revascularization			2			0			2	
		**Acute myocardial infarction, unspecified, type 2 (I21.9 + I98.2)**					3			0			3
			**Revascularization**			0			0			0	
				Percutaneous coronary intervention	0			0			0		
				Coronary artery bypass grafting	0			0			0		
			No revascularization			0			0			0	
	**Revascularization without acute coronary syndrome diagnosis**						6			1			7
		Percutaneous coronary intervention					6			1			7
		Coronary artery bypass grafting					0			0			0
	Cardiovascular death						0			0			0

^a^Data were derived from the electronic health record.

^b^7 days: outcome at discharge or ≤7 days from the emergency department visit.

^c^30 days: outcome after discharge or >7 days, and ≤30 days from the emergency department visit.

**Table 5:** Primary ICD-10^a^ code and diagnosis for the 754 patients discharged home.^b^

ICD-10 code	Diagnosis	Values, n (%)
R07	Pain in throat and chest	574 (76.1)
R00-09 (excluding R07)	Symptoms and signs involving the circulatory and respiratory systems	57 (7.6)
R10-49	Symptoms and signs involving other organ systems	23 (3.1)
I48	Atrial fibrillation and flutter	16 (2.1)
R50-69	General symptoms and signs	14 (1.9)
M79	Other soft tissue disorders, not elsewhere classified	11 (1.5)
K29.7, K30.9, and K21.9	Gastritis, unspecified; functional dyspepsia; gastro-esophageal reflux disease without esophagitis	10 (1.3)
I20.1-9	Angina pectoris	4 (0.5)
I26.9	Pulmonary embolism without mention of acute cor pulmonale	3 (0.4)
J18.9	Pneumonia, unspecified	2 (0.3)
J45.9	Asthma, unspecified	2 (0.3)
I30.9	Acute pericarditis, unspecified	1 (0.1)
I63.9	Cerebral infarction, unspecified	1 (0.1)
J44.1	Chronic obstructive pulmonary disease with acute exacerbation, unspecified	1 (0.1)
N/A^c^	Other nonsevere diagnosis	35 (4.6)

^a^ICD-10: International Statistical Classification of Diseases and Related Health Problems, 10th Revision.

^b^Diagnoses reported by the physician in the electronic health record for patients with a chief complaint of chest pain who were discharged home (n=754), either directly from the emergency department (n=462) or via the day-care unit (n=292).

^c^N/A: not applicable.

**Table 6:** Primary discharge ICD-10^a^ code and diagnosis for hospitalized patients (N=236).^b^

ICD code	Diagnosis			Values, n (%)
I20.0, I21	Acute coronary syndrome^c^			62 (26.3)
I20.1-9	Angina pectoris			14 (5.9)
I25.9	Chronic ischemic heart disease, unspecified			8 (3.4)
I05.0	Mitral stenosis			1 (0.4)
I10.9	Essential (primary) hypertension			1 (0.4)
I26-I28	Pulmonary heart disease and diseases of pulmonary circulation			6 (2.5)
I30-I52	**Other forms of heart disease**			35 (14.8)
		I30: Acute pericarditis	2 (5.7)	
		I35: Nonrheumatic aortic valve disorders	3 (8.6)	
		I40: Acute myocarditis	5 (14.3)	
		I42: Cardiomyopathy	1 (2.9)	
		I44: Atrioventricular and left bundle-branch block	1 (2.9)	
		I47: Paroxysmal tachycardia	2 (5.7)	
		I48: Atrial fibrillation and flutter	10 (28.6)	
		I49: Other cardiac arrhythmias	3 (8.6)	
		I50: Heart failure	8 (22.9)	
I95-I99	Other and unspecified disorders of the circulatory system			1 (0.4)
J09-J18	Influenza and pneumonia			4 (1.7)
J20	Acute bronchitis			1 (0.4)
J44.1	Chronic obstructive pulmonary disease with acute exacerbation, unspecified			1 (0.4)
K00-93	Diseases of the digestive system			7 (3.0)
R07.4	Chest pain, unspecified			64 (27.1)
R00-09 (excluding R07.4)	Symptoms and signs involving the circulatory and respiratory systems			11 (4.7)
R10-49	Symptoms and signs involving other organ systems			1 (0.4)
R50-69	General symptoms and signs			2 (0.8)
N/A^d^	Other nonsevere diagnosis singularly reported			17 (7.2)

^a^ICD-10: International Statistical Classification of Diseases and Related Health Problems, 10th Revision.

^b^Data reported by the physician in the electronic health record for patients with a chief complaint of chest pain who were admitted from the ED (n=236). Of these, 15 patients were admitted to a noncardiac ward.

^c^Primary diagnosis of I20.0 (unstable angina pectoris) or I21 (acute myocardial infarction).

^d^N/A: not applicable.

### Diagnostic Accuracy for a MACE or ACS

A clinically decisive score could be calculated using CHT-acquired data in 751 out of 1000 (75.10%; D-HEART), 727 out of 1000 (72.70%; HEART), 735 out of 1000 (73.50%; EDACS-ADP), and 838 out of 1000 (83.80%; T-MACS) patients (Figure 1). For D-HEART, 24 of 751 (3.2%) patients were included despite insufficient CHT data, based on rule-in troponin findings. Patients with insufficient CHT data to calculate the risk scores were generally younger and, for all risk scores except T-MACS, more often female (Table S2 in Multimedia Appendix 1). No adverse events occurred during the CHT interviews.

**Figure 2:**
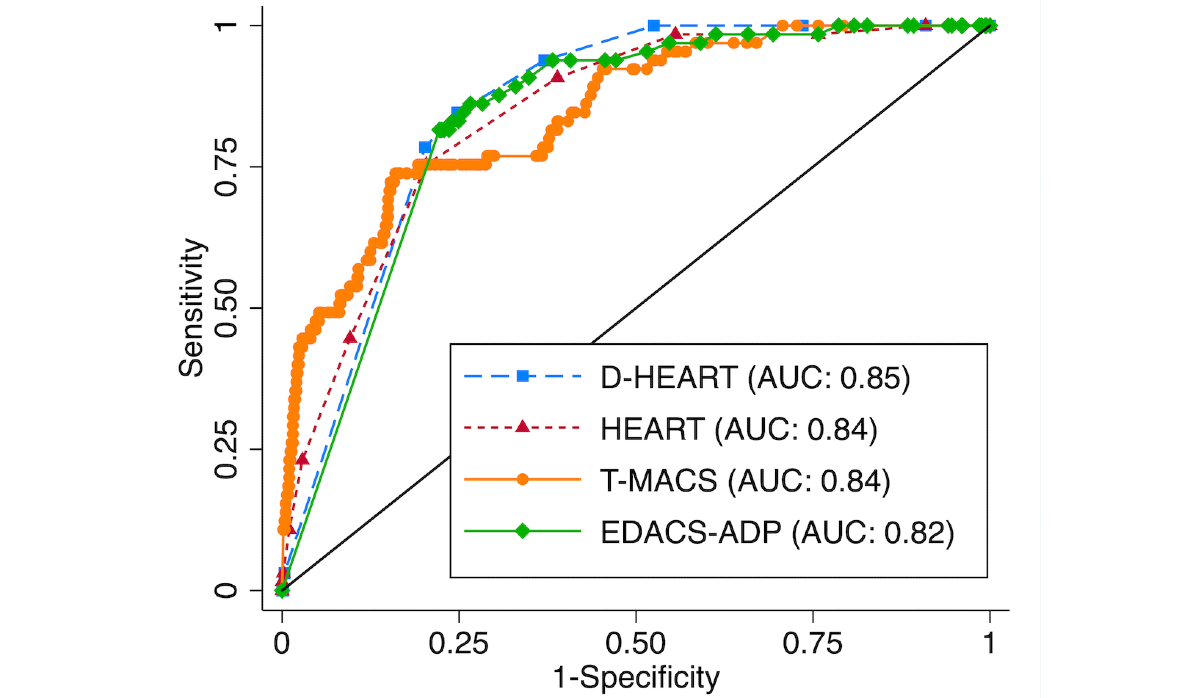
Receiver operating curves for HEART, D-HEART, EDACS-ADP and T-MACS for a 30-day major adverse cardiac event. AUC: area under the receiver operating curve; D-HEART: Danderyd HEART; EDACS-ADP: Emergency Department Assessment of Chest Pain Score combined with an Accelerated Diagnostic Protocol; HEART: History, ECG, Age, Risk Factors, and Troponin; T-MACS: Troponin-only Manchester Acute Coronary Syndrome.

For MACE, all 4 risk scores demonstrated high NPVs of 0.99 (95% CI 0.97-1.00). Sensitivity ranged from 0.91 (95% CI 0.81-0.97; HEART) to 0.97 (95% CI 0.90-1.00; T-MACS <0.02). The MACE miss rate among patients classified as low risk was 2 out of 286 (0.70%; T-MACS), 4 out of 416 (1.0%; D-HEART), 4 out of 346 (1.2%; EDACS-ADP), and 6 out of 406 (1.5%; HEART). The proportion of patients ruled out was highest for HEART (406/727, 55.8%) and D-HEART (416/751, 55.4%), intermediate for EDACS-ADP (346/744, 46.5%), and lowest for T-MACS (286/838, 34.1%; Table 7). Using T-MACS with a threshold of <0.05 for low risk yielded a sensitivity, specificity, PPV, and NPV of 0.89 (95% CI 0.78-0.95), 0.59 (95% CI 0.55-0.62), 0.16 (95% CI 0.12-0.20), and 0.98 (95% CI 0.97-0.99), respectively. Across CHT-derived risk scores, diagnostic performance for ACS was comparable to that for MACE (Table S3 in Multimedia Appendix 1). T-MACS <0.02 provided higher sensitivity and NPV but lower specificity compared with T-MACS <0.05 (Table S4 in Multimedia Appendix 1). No differences in AUC between the risk scores were observed for either MACE or ACS (Figure 2 and Table S5 in Multimedia Appendix 1). Excluding the 3 patients diagnosed with unstable angina pectoris who did not undergo revascularization (Table S6 in Multimedia Appendix 1), or the patient who died from a noncardiac cause, had a negligible effect on the results.

**Table 7:** Rule-out performance of risk scores populated with data derived from computerized history taking for a major adverse cardiac event within 30 days.^a^

Risk score	Sensitivity (95% CI)	Specificity (95% CI)	Positive predictive value (95% CI)	Negative predictive value (95% CI)	Miss rate, n/N (%)	Patients ruled out, n/N (%)
D-HEART^b^ (n=751)	0.94 (0.86-0.98)	0.60 (0.57-0.64)	0.19 (0.15-0.24)	0.99 (0.98-1.00)	4/416 (0.96)	416/751 (55.39)
HEART^c^ (n=727)	0.91 (0.81-0.97)	0.61 (0.57-0.64)	0.19 (0.15-0.23)	0.99 (0.97-1.00)	6/406 (1.48)	406/727 (55.85)
EDACS-ADP^d^ (n=744)	0.94 (0.86-0.98)	0.51 (0.48-0.55)	0.17 (0.13-0.21)	0.99 (0.97-1.00)	4/346 (1.16)	346/744 (46.51)
T-MACS^e^ (n=838)	0.97 (0.90-1.00)	0.37 (0.34-0.39)	0.12 (0.10-0.15)	0.99 (0.98-1.00)	2/286 (0.70)	286/838 (34.13)

^a^When T-MACS with a threshold of <0.05 for low risk was applied, the numbers of true positives, false positives, false negatives, and true negatives were 55, 294, 7, and 418, respectively. Sensitivity, specificity, PPV, and NPV were 0.89 (95% CI 0.78-0.95), 0.59 (95% CI 0.55-0.62), 0.16 (95% CI 0.12-0.20), and 0.98 (95% CI 0.97-0.99), respectively.

^b^D-HEART: Danderyd HEART.

^c^HEART: History, ECG, Age, Risk Factors, and Troponin.

^d^EDACS-ADP: Emergency Department Assessment of Chest Pain Score combined with an Accelerated Diagnostic Protocol.

^e^T-MACS: Troponin-only Manchester Acute Coronary Syndrome.

### D-HEART Versus HEART

When comparing the NPV (95% CI) for ruling out MACE and ACS, no difference (*P*<.001) was found between the D-HEART and HEART scores: 0.99 (0.98-1.00) versus 0.99 (0.97-1.00) for MACE, and 0.99 (0.98-1.00) versus 0.99 (0.97-1.00) for ACS, respectively. Similarly, no difference in diagnostic accuracy was observed when comparing the AUCs (Table S5 in Multimedia Appendix 1).

### Risk Reclassification for MACE or ACS

Of the 1000 participants, 528 were admitted, with 203 admitted to the ward and 325 to the day-care unit. Of the day-care unit admissions, 33 were subsequently admitted to the ward, and 292 were discharged home (Table 3). If CHT data had been used instead of standard management to calculate D-HEART, HEART, and EDACS-ADP, there would have been a net reclassification from “nonlow risk” to “low risk” in 89 (16.9%), 89 (16.9%), and 31 (5.9%) cases, respectively. However, due to lower specificity, use of T-MACS would instead have resulted in 91 (17.2%) patients being net reclassified from “low risk” to “nonlow risk.” These reclassifications correspond to a net shift in MACE or ACS outcomes of +2 (D-HEART), +4 (HEART), +2 (EDACS-ADP), and 0 (T-MACS; Table 8).

**Table 8:** Reclassification and MACE^a^ or ACS^b^ within reclassified groups if risk scores populated with computerized history-taking data had been used for the disposition of patients instead of physician management.^c^

Reclassification			D-HEART^d^		HEART^e^		EDACS-ADP^f^		T-MACS^g^
**Low to nonlow risk, n**			93		97		128		220
	MACE or ACS, n	1		1		2		1	
**Nonlow risk to low risk, n**			182		186		159		129
	MACE or ACS, n	3		5		4		1	
**Difference^h^**			89		89		31		–91
	MACE or ACS, n	+2		+4		+2		0	
Proportion of admissions, n/N (%)			89/528 (–16.9%)		89/528 (–16.9%)		31/528 (–5.9%)		91/528 (+17.2%)

^a^MACE: major adverse cardiac event.

^b^ACS: acute coronary syndrome.

^c^Admission to the ward or day-care unit (n=528) was considered a proxy for nonlow risk, and discharge to home (n=462) a proxy for low risk. Data for 1 participant were missing, and 9 left the ED without seeing a physician.

^d^D-HEART: Danderyd HEART.

^e^HEART: History, ECG, Age, Risk Factors, and Troponin.

^f^EDACS-ADP: Emergency Department Assessment of Chest Pain Score combined with an Accelerated Diagnostic Protocol.

^g^T-MACS: Troponin-only Manchester Acute Coronary Syndrome.

^h^The difference indicates the n and % change in admissions.

## Discussion

### Principal Findings

This study shows that CHT can be used to calculate 4 risk scores in a majority (727-838/1000, 72.70%-83.80%) of patients presenting with acute chest pain, with good diagnostic performance for ruling out 30-day 3-point MACE or ACS, yielding NPVs of 0.99 (95% CI 0.97-1.00) for both outcomes. Sensitivities ranged from 91% to 97%, implying that, out of 100 patients, 3-9 with MACE might be misclassified. While this may initially appear concerning, the clinically more relevant miss rate among patients classified as low risk remained low for D-HEART (4/416, 0.96%) and T-MACS (2/286, 0.70%), aligning with commonly accepted safety thresholds for MACE (NPV ≥0.99 and miss rate <1%). By contrast, the HEART score (6/406, 1.5%) and EDACS-ADP (4/346, 1.2%) showed higher miss rates [16,48]. However, the lower bounds of the 95% CIs for sensitivity allow miss rates exceeding accepted rule-out safety thresholds; therefore, these findings warrant cautious interpretation.

In addition, CHT-derived chest pain risk scores reclassified a substantial proportion (up to 89/528, 16.9%) of patients admitted with acute chest pain, initially managed as suspected ACS, from “nonlow risk” to “low risk” at the time of initial ED assessment, potentially facilitating a decision for discharge rather than hospital admission. However, hospital admission may be driven by factors other than ACS risk, and reclassification to low risk does not necessarily eliminate the need for admission; thus, the aforesaid figure (ie, 89/528, 16.9%) should be viewed as a theoretical maximum, and real-world discharge gains may be substantially lower. The attendant trade-off is an increased miss rate of MACE or ACS among reclassified patients. In our study, the number of missed events (0-4 events, depending on the risk score used) was similar to that observed under standard management (2 events). While our findings indicate that CHT is a promising tool for risk score determination, further studies are warranted to validate these results and to ascertain safety.

### Comparison With Prior Work

Our findings on diagnostic accuracy align with multiple validation studies in which traditional physician-acquired medical histories were used, demonstrating the efficacy of risk scores in safely ruling out MACE in the ED [7-10,31,49]. However, this appears to be the first study to report on the performance of an automated method for collecting the medical history required to calculate these risk scores. The assessed risk scores (D-HEART, HEART, EDACS-ADP, and T-MACS) showed similar performance for ruling out 30-day MACE or ACS. Our findings are consistent with prior work. A meta-analysis of 25 studies (n=25,266) reported a pooled sensitivity of 0.96 (95% CI 0.93-0.98) and an NPV of 0.99 (95% CI 0.98-0.99) for HEART [7]. A meta-analysis of the EDACS score (8 studies, n=11,578) reported a pooled sensitivity of 0.95 (95% CI 0.90-0.99) but did not specifically report NPV [50]. External validation of EDACS-ADP in a North American cohort (n=763) demonstrated a sensitivity and NPV of 1.00 (95% CI 0.94-1.00) and 1.00 (95% CI 0.99-1.00), respectively [8]. For T-MACS, secondary analyses of 4 UK cohort studies (n=1459) reported a sensitivity and NPV of 0.98 (95% CI 0.95-1.00) and 0.99 (95% CI 0.98-1.00), respectively [32]. However, the HEART score, which has undergone more extensive validation, consistently demonstrates good performance and is endorsed by international societies [5,51].

The only methodological difference between HEART and D-HEART in our study was the use of the ESC 0/1-hour troponin algorithm in D-HEART. Thus, the comparison primarily reflects the added value of serial troponin testing rather than differences related to CHT. D-HEART demonstrated equivalent performance for MACE or ACS rule out compared with the original HEART score using only a single baseline troponin measurement. This could be interpreted as support for using the simpler HEART score without repeated troponin measurements. However, this contrasts with findings showing that the original HEART score has somewhat lower diagnostic accuracy than a combination of the HEART score and a 0/1-hour troponin algorithm [52]. As the HEART score was primarily developed and validated for safe rule out of MACE, it is reasonable to assume that integrating a 0/1-hour algorithm enhances rule-in capability, as previously suggested [53]. Accordingly, the superior safety profile observed for D-HEART in this study is likely driven by the integration of serial troponin measurements rather than the CHT-derived history component itself. Nevertheless, the absolute difference in missed events was small (4 vs 6), and neither NPV nor AUC differed significantly between the scores. Thus, the difference in performance between the 2 scores may not be statistically significant. Taken together, D-HEART appears to be the most promising option for CHT-based risk stratification; however, future studies are needed to confirm these findings.

Our findings show an AUC of 0.82-0.85 and an NPV of 0.98-0.99, depending on the risk score used. While there is no universal benchmark for AUC or NPV in risk stratification, an AUC of 0.8-0.9 is generally considered excellent, and >0.9 outstanding [54]. Interpretation of NPV varies by clinical context; for MACE or ACS, a high NPV is desirable for safe rule out [55]. Overall, our findings suggest that risk calculation using CHT data performs at least as well as traditional history taking. It is reassuring that all scores reproduced similar results. However, further refinement of CHT data collection could potentially enhance MACE rule-out capability. Notably, a strategy in which CHT data are used for risk calculation requires further validation. Importantly, risk score algorithms should always be considered as decision-support tools and used in complement to physician expertise.

### Clinical Implications

A significant fraction (up to 89/528, 16.9%) of participants were reclassified from “nonlow risk” to “low risk” when using D-HEART, HEART, and EDACS-ADP populated with CHT data, compared with standard management. These findings align with those of an external validation study, which reported a reduction in hospital admissions for patients with acute chest pain of approximately 15% following implementation of the HEART score [56]. Improvements in classification may reduce anxiety, unnecessary examinations, and health care resource use [57]. However, a large Dutch multicenter randomized controlled trial observed a limited impact of risk score calculations on resource utilization, attributing this effect to nonadherence to management recommendations [58]. Notably, when using T-MACS, more patients would be reclassified from “low risk” to “nonlow risk,” potentially leading to increased admissions (91/528, +17.2%) compared with standard care, due to its lower specificity. Applying a higher threshold for T-MACS (<0.05 for “low risk”) increases specificity but results in a lower NPV, supporting the conclusion that this is not the preferred score for patient disposition. Further studies on optimal CHT implementation and its impact on resource utilization and quality of care in chest pain management are warranted.

This study extends our previous reports demonstrating substantial agreement between data collected using CHT and standard history taking for risk score assessment [30]. However, for the HEART score, the History component was derived from predefined patient-reported CHT criteria rather than physician gestalt (ie, physician’s subjective clinical judgment), as applied in the original derivation studies. Given the previously demonstrated low-to-moderate agreement between CHT and EHR data for chest pain characteristics [30], this substitution may affect construct validity and limit direct comparability with physician-acquired HEART scores. At the same time, prior work suggests that CHT captures more complete and detailed symptom data than are routinely documented by physicians in the EHR, which may partly explain the observed discrepancies. Our previous studies also showed that risk score calculation was feasible to a much greater extent when using CHT-collected data compared with physician-reported EHR data (74%-83% vs 10%-31%, depending on the risk score used), and that a HEART score excluding the troponin variable (HEART score) can effectively predict clinical outcomes [54]. This analysis underscores the broader utility of CHT in facilitating the calculation of established risk scores commonly used in clinical practice. Moreover, this study introduces the concept of a clinically decisive risk score, defined as reaching a specific threshold for “nonlow risk” (eg, D-HEART≥4 or T-MACS≥0.02), to better reflect how these risk stratification tools are applied in practice.

The time required to obtain sufficient data for risk score calculation using CHT (median 23 minutes) may appear long. However, the intention of CHT was not to replace physician history taking, but rather to use waiting time in the ED to collect and supplement crucial information for risk stratification. Notably, the median length of stay for low- to intermediate-risk patients (ie, RETTS orange, yellow, green, and blue) in the ED at the time of the study was approximately 4 hours, suggesting that CHT can be completed without disrupting workflow. Nevertheless, future developments of CHT should focus on shortening interview duration and adapting content to the clinical context and urgency. Moreover, although physician-interpreted ECGs were used by design to isolate the effect of CHT on chest pain management, the incorporation of automated ECG analysis may further enhance efficiency.

Considering the high volume of patients presenting to the ED with chest pain, automating risk stratification through CHT could streamline the ED pathway: first, by ensuring complete and structured capture of medical history and risk factors; second, by reducing physician time spent on data gathering; third, by providing real-time support for clinical decision-making; and finally, by enabling faster and safer disposition decisions that identify patients suitable for discharge and reduce resource use.

### Strengths and Limitations

Key strengths of this study include its prospective cohort design, a large and representative ED chest pain population, evaluation under real-world conditions in the typical clinical environment in which the tool would be used, and reliable outcomes defined by strict, well-established criteria. The consistency of the findings across multiple validated risk scores underscores the robustness of the results. Furthermore, the generic layout of the CHT software, which is not limited to cardiology or the ED setting, suggests that these findings may apply to other clinical contexts. Finally, the study was initiated and conducted within an academic setting, without any commercial interests.

However, several limitations warrant consideration. First, the single-center design introduces a risk of selection bias, potentially limiting population diversity. In addition, the use of a convenience sample comprising a minority of eligible chest pain presentations may limit external validity, as enrolled patients may be more compliant, clinically stable, or technologically literate than the overall ED population. This bias may have been further exacerbated by the exclusion of patients unable to complete CHT and by recruitment occurring mainly during office hours and evenings, due to research staff availability. However, the proportion of patients with chest pain presenting at night was small, and the demographics of study participants were similar to those of the overall ED chest pain population, reducing the likelihood of major systematic differences. Nevertheless, our findings should be interpreted with caution, and future large multicenter studies are warranted to ascertain the generalizability of these results to other health care settings. Second, variability in physician performance and the unique ED setup, which includes a dedicated cardiology unit and a day-care unit staffed by cardiologists, may lead to an underestimation of CHT performance compared with settings with less specialist expertise in the ED. At the same time, this organizational structure may limit the generalizability of our findings to more diverse clinical environments. Third, risk score determination using CHT data was not feasible for approximately one-quarter of participants. We have previously shown that premature termination of the CHT interview was associated with ED discharge and fatigue, most commonly among older adults (≥70 years), individuals born outside Europe, and retirees, thereby limiting conclusions for these groups [22]. In our analysis, however, patients with insufficient CHT data were significantly younger than those with a clinically decisive score (mean age 51.0 vs 56.2 years; see Table S2 in Multimedia Appendix 1), likely reflecting the larger proportion of adults aged 18-69 years in the study population and their higher frequency of interview termination, often associated with early discharge [22]. Nevertheless, diagnostic performance was assessed only in patients who provided sufficient CHT data for risk score determination, which may have overestimated real-world performance. This finding underscores the need for a more user-friendly tool to reduce missing data in routine care. Fourth, ACS outcomes were adjudicated by a single board-certified cardiologist, which may introduce adjudication bias compared with evaluation by 2 or more independent specialists. However, Swedish discharge ICD codes have a high PPV (85%-95%) for acute myocardial infarction and related diagnoses, supporting the reliability of the outcome classification [45,46]. Further, outcomes were retrieved through passive review of the shared EHR system in Region Stockholm; therefore, events occurring outside the region may not have been captured, potentially leading to an overestimation of the NPV. Mortality status is, however, captured through linkage between the regional EHR system and the national population registry. The proportion of nonfatal events is likely small, as routine follow-up after a MACE is systematically conducted at the patient’s home hospital, even when the event occurs outside the region. Fifth, given that patients with MACE were significantly older than those without (ie, non-MACE; 67.6 vs 53.7 years), there is a risk of spectrum bias if CHT fails to capture data from patients at the highest risk. In our cohort, however, insufficient CHT data were not more common among older patients (Table S2 in Multimedia Appendix 1), reducing concerns that missing CHT data systematically occurred in older or higher-risk individuals. Sixth, inclusion in the study could occur either before or after the patient had been evaluated by a physician. This may have introduced recall bias, as patients might provide different responses when reporting their history a second time. The effect of interview order on patient responses was not assessed and therefore remains an unmitigated limitation. Seventh, patient disposition reflects not only the medical history obtained by the physician but also additional information available at the time of assessment, including ECG findings, blood test results, and prior EHR documentation. These factors likely influenced clinical decision-making, thereby limiting direct comparability with CHT-derived risk scores. However, this approach was intentionally chosen to evaluate the potential impact of CHT within current clinical management. Future multimodal studies should aim to incorporate such data directly into risk stratification models. Eighth, the primary focus of this study was the safe rule out of MACE or ACS using a clinically decisive risk score. This implies that once patients exceed the threshold for “nonlow risk” (eg, HEART ≥4), they are considered ruled in. However, some patients within this clinically decisive risk group who did not complete the CHT interview might have achieved even higher total risk scores. Consequently, our findings may be more robust for rule out than for rule in. Conversely, excluding patients with insufficient CHT data who also did not meet ECG or troponin criteria for “nonlow risk” may have resulted in the systematic exclusion of a specific patient subset, potentially leading to an overestimation of NPVs. Accordingly, interpretation of both rule-out and rule-in performance should account for these methodological limitations, and further evaluation across the full risk spectrum is warranted. Finally, this study carries a potential risk of verification bias. We believe this risk to be minimal, as patients were managed according to standard care protocols independent of CHT-derived risk scores, which were not available to the treating physicians during clinical decision-making.

### Future Directions

Future research on CHT in chest pain risk stratification should include customizing CHT for the ED setting to reduce interview time, extending its use to early prehospital triage, and broadening its scope to support diagnosis of a wider array of acute conditions, such as pulmonary embolism, aortic dissection, and other noncardiac causes of chest pain. In addition, future studies should explore the integration of multimodal variables into risk stratification models, and the incorporation of multiple languages into the CHT software could enable history taking in patients’ native languages.

### Conclusions

Automated medical history taking using CHT can generate reliable risk scores in the majority of patients presenting with acute chest pain, with good diagnostic performance for ruling out 30-day 3-point MACE (ie, ACS diagnosis, revascularization, or cardiovascular death) and ACS. CHT-derived risk scores performed comparably to previously validated physician-acquired scores, and accepted safety thresholds were met by D-HEART and T-MACS, whereas the HEART score and EDACS-ADP showed values slightly above the commonly accepted <1% threshold; however, given the wide sensitivity CIs, these findings should be interpreted with caution. Compared with the HEART score, the improved safety of D-HEART appears to be primarily attributable to the incorporation of serial 0/1-hour troponin testing rather than the CHT-derived history itself. Application of CHT-derived risk scores indicated that 89 of the 528 (16.9%) patients could potentially be reclassified from “nonlow risk” to “low risk” at the time of initial ED assessment, which may support discharge decisions in selected patients, although admission may still be required for reasons unrelated to ACS. Consistent with prior chest pain risk score studies, any gains in discharge rates must be balanced against the risk of missed events among reclassified patients. Among the scores assessed, D-HEART appears most promising for CHT-based risk stratification. However, further large multicenter studies are warranted to confirm these findings before CHT can be established in routine clinical practice.

## Appendix

Multimedia Appendix 1 Additional analysis.

## Abbreviations

ACS: acute coronary syndrome
AUC: area under the receiver operating characteristic curve
CHT: computerized history taking
CLEOS-CPDS: Clinical Expert Operating System Chest Pain Danderyd Study
D-HEART: Danderyd HEART
ECG: electrocardiogram
ED: emergency department
EDACS-ADP: Emergency Department Assessment of Chest Pain Score combined with an Accelerated Diagnostic Protocol
EHR: electronic health record
ESC: European Society of Cardiology
HEART: History, ECG, Age, Risk Factors, and Troponin
hs-cTn: high-sensitivity cardiac troponin
ICD-10: International Statistical Classification of Diseases and Related Health Problems, 10th Revision
MACE: major adverse cardiac event
NPV: negative predictive value
PPV: positive predictive value
RETTS: Rapid Emergency Triage and Treatment System
STARD: Standards for Reporting Diagnostic Accuracy
T-MACS: Troponin-only Manchester Acute Coronary Syndrome

## Data Availability

The datasets used or analyzed during this study are available from the corresponding author (HB) on reasonable request.
