# Supplementary material for: Computerized Self-Reported Medical History Taking to Support Early Rule Out of Major Adverse Cardiac Events in Patients With Acute Chest Pain: Post Hoc Analysis of the CLEOS-CPDS Prospective Cohort Study
Source: J Med Internet Res. 2026 Feb 11;28:e76087. doi: 10.2196/76087 (PMC12936665; doi:10.2196/76087)
Supplement: Multimedia Appendix 1 [file jmir_v28i1e76087_app1.docx]

Computerized Self-Reported Medical History Taking to Support Early Rule Out of Major Adverse Cardiac Events in Patients With Acute Chest Pain: A Post-Hoc Analysis of the CLEOS-CPDS Prospective Cohort Study

**Supplementary material**

**Supplementary Tables**

Supplementary Table 1. Electrocardiogram reports and interpretations. 2

Supplementary Table 2. Comparison of demographics between patients with a decisive risk score and those excluded due to insufficient CHT data for risk score calculation. 3

Supplementary Table 3. Performance of risk scores populated with data derived from computerized history taking for an acute coronary syndrome within 30 days 4

Supplementary Table 4a. Performance of T-MACS using the “low” threshold of <0.05 and populated with data derived from computerized history taking for a major adverse cardiac event within 30 days 5

Supplementary Table 4b. Performance of T-MACS using the “low” threshold of <0.05 and populated with data derived from computerized history taking for an acute coronary syndrome within 30 days 5

Supplementary Table 5. AUC of risk scores populated with data derived from computerized history taking for a major adverse cardiac event within 30 days 6

Supplementary Table 6. Performance of risk scores populated with data derived from computerized history taking for a 30-day MACE, excluding the three patients with unstable angina pectoris who did not undergo revascularization 7

## Supplementary Table 1. Electrocardiogram reports and interpretations.

| **Reported ECG interpretation** | **Patients, n (%)** |
| --- | --- |
| **Considered as (1) 2 points for HEART score, (2) “not low risk” with EDACS (new ischemia on ECG) and (3) ECG diagnostic for ischemia in T-MACS. All previously unknown findings.** | |
| ST elevation (not widespread) | 5 (0.5) |
| ST elevation (widespread) | 3 (0.3) |
| ST depression | 15 (1.5) |
| Moderate STT changes | 1 (0.1) |
| Inverted T waves | 19 (1.9) |
| STT changes (not specified) | 7 (0.7) |
| Left bundle branch block  New onset, 3 (0.3)  Not specified/unknown if new onset, 6 (0.6) | 9 (0.9) |
| Right bundle branch block  New onset, 3 (0.3)  Not specified/unknown if new onset, 4 (0.4) | 7 (0.7) |
|  |  |
| **Considered as 1 point for HEART score** | |
| Nonspecific STT wave changes (unknown) | 91 (9.1) |
| Known bifascicular block | 1 (0.1) |
| Known left bundle branch block | 13 (1.3) |
| Known right bundle branch block | 15 (1.5) |
|  |  |
| **Not considered as ECG with new ischemia. All previously known findings.** | |
| Nonspecific STT wave changes | 3 (0.3) |
| Moderate STT changes | 1 (0.1) |
| ST elevation | 1 (0.1) |
| ST depression | 3 (0.3) |
| Inverted T waves | 7 (0.7) |

Values represent numbers (%) for 1000 patients. If no ECG could be found in the electronic health record (n=6) the ECG was considered as missing and calculations of risk scores not possible. ECG interpretation was systematically categorized based on rhythm, QRS formation and STT changes. ECGs without any reported abnormal QRS or STT changes, by either physician or computer interpretation, were considered negative. Information about known pre-existing abnormalities was recorded if mentioned. Isolated negative T-waves in leads V1, V2, III, aVL or aVF were not considered as pathological. STT deviations in ECGs with signs of left ventricular hypertrophy were considered related to this condition unless explicitly identified as pathological. ECG: electrocardiogram

**Supplementary Table 2.** Comparison of demographics between patients with a decisive risk score and those excluded due to insufficient CHT data for risk score calculation.

|  | **All** | **Patients with a decisive risk score** | **Patients with insufficient CHT data** | ***P* value** |
| --- | --- | --- | --- | --- |
| **D-HEART**, n | 972 | 751 | 221 |  |
| Age, years | 55.0 (±17.1) | 56.2 (±17.2) | 51.0 (±16.1) | <.001 |
| Sex (females) | 441 (45.4) | 323 (43.0) | 118 (53.4) | .01 |
| **HEART**, n | 971 | 727 | 244 |  |
| Age, years | 55.0 (±17.1) | 55.7 (±16.9) | 52.9 (±17.5) | .03 |
| Sex (females) | 440 (45.3) | 311 (42.8) | 129 (52.9) | .01 |
| **EDACS-ADP**, n | 1000 | 735 | 265 |  |
| Age, years | 54.7 (±17.2) | 56.5 (±17.2) | 49.9 (±16.1) | <.001 |
| Sex (females) | 456 (45.6) | 313 (42.6) | 143 (54.0) | .001 |
| **T-MACS**, n | 1000 | 838 | 162 |  |
| Age, years | 54.7 (±17.2) | 55.9 (±17.1) | 48.9 (±16.2) | <.001 |
| Sex (females) | 456 (45.6) | 378 (45.1) | 78 (48.1) | .48 |

Data are presented as mean values ± SD or n (%), as appropriate. CHT: computerized history-taking.

**Supplementary Table 3.** Performance of risk scores populated with data derived from computerized history taking for an acute coronary syndrome within 30 days.

|  | **Truepos** | **False pos** | **False neg** | **True neg** | **Sensitivity** | **Specificity** | **PPV** | **NPV** |
| --- | --- | --- | --- | --- | --- | --- | --- | --- |
| **D-HEART** | 58 | 277 | 4 | 412 | 0.94  (0.84–0.98) | 0.60  (0.56–0.64) | 0.17  (0.13–0.22) | 0.99  (0.98–1.00) |
| **HEART** | 53 | 268 | 6 | 400 | 0.90  (0.79–0.96) | 0.60  (0.56–0.64) | 0.17  (0.13–0.21) | 0.99  (0.97–1.00) |
| **EDACS-ADP** | 59 | 330 | 4 | 342 | 0.94  (0.85–0.98) | 0.51  (0.47–0.55) | 0.15  (0.12–0.19) | 0.99  (0.97–1.00) |
| **T-MACS** | 61 | 491 | 2 | 284 | 0.97  (0.89–1.00) | 0.37  (0.33–0.40) | 0.11  (0.09–0.14) | 0.99  (0.98–1.00) |

pos: positive. neg: negative. PPV: positive predictive value. NPV: negative predictive value.

Data are presented as n or probability with 95% confidence interval.

**Supplementary Table 4a.** Performance of T-MACS using the “low” threshold of <0.05 and populated with data derived from computerized history taking for a *major adverse cardiac event* within 30 days.

|  | **Truepos** | **False pos** | **False neg** | **True neg** | **Sensitivity** | **Specificity** | **PPV** | **NPV** |
| --- | --- | --- | --- | --- | --- | --- | --- | --- |
| **T-MACS<.02** | 67 | 485 | 2 | 284 | 0.97  (0.90–1.00) | 0.37  (0.34–0.39) | 0.12  (0.10–0.15) | 0.99  (0.98–1.00) |
| **T-MACS<.05** | 55 | 294 | 7 | 418 | 0.89  (0.78–0.95) | 0.59  (0.55–0.62) | 0.16  (0.12–0.20) | 0.98  (0.97–0.99) |

pos: positive. neg: negative. PPV: positive predictive value. NPV: negative predictive value.

Data are presented as n or probability with 95% confidence interval.

**Supplementary Table 4b.** Performance of T-MACS using the “low” threshold of <0.05 and populated with data derived from computerized history taking for an *acute coronary syndrome* within 30 days.

|  | **Truepos** | **False pos** | **False neg** | **True neg** | **Sensitivity** | **Specificity** | **PPV** | **NPV** |
| --- | --- | --- | --- | --- | --- | --- | --- | --- |
| **T-MACS<.02** | 61 | 491 | 2 | 284 | 0.97  (0.89–1.00) | 0.37  (0.33–0.40) | 0.11  (0.09–0.14) | 0.99  (0.98–1.00) |
| **T-MACS<.05** | 52 | 297 | 6 | 419 | 0.90  (0.79–0.96) | 0.59  (0.55–0.62) | 0.15  (0.11–0.19) | 0.99  (0.97–1.00) |

pos: positive. neg: negative. PPV: positive predictive value. NPV: negative predictive value.

Data are presented as n or probability with 95% confidence interval.

**Supplementary Table 5.** AUC of risk scores populated with data derived from computerized history taking for a major adverse cardiac event within 30 days.

|  | **Observations** | **AUC** | **Standard error** | **95% CI** |
| --- | --- | --- | --- | --- |
| **D-HEART** | 751 | 0.85 | 0.02 | 0.82–0.88 |
| **HEART** | 727 | 0.84 | 0.02 | 0.80–0.89 |
| **EDACS-ADP** | 735 | 0.82 | 0.02 | 0.78–0.86 |
| **T-MACS** | 838 | 0.84 | 0.03 | 0.79–0.89 |

No difference found for AUC between the risk scores. AUC: area under the receiver operating curve; CI: confidence interval.

**Supplementary Table 6.** Performance of risk scores populated with data derived from computerized history taking for a 30-day MACE, excluding the three patients with unstable angina pectoris who did not undergo revascularization.

|  | **Truepos** | **False pos** | **False neg** | **True neg** | **Sensitivity** | **Specificity** | **PPV** | **NPV** |
| --- | --- | --- | --- | --- | --- | --- | --- | --- |
| **D-HEART**  **(n=748)** | 62 | 270 | 4 | 412 | 0.94  (0.85–0.98) | 0.60  (0.57–0.64) | 0.19  (0.15–0.23) | 0.99  (0.98–1.00) |
| **HEART**  **(n=725)** | 58 | 261 | 6 | 400 | 0.91  (0.81–0.97) | 0.61  (0.57–0.64) | 0.18  (0.14–0.23) | 0.99  (0.97–1.00) |
| **EDACS-ADP**  **(n=741)** | 60 | 323 | 4 | 354 | 0.94  (0.85–0.98) | 0.52  (0.49–0.56) | 0.16  (0.12–0.20) | 0.99  (0.97–1.00) |
| **T-MACS**  **(n=835)** | 64 | 485 | 2 | 284 | 0.97  (0.90–1.00) | 0.37  (0.34–0.41) | 0.12  (0.09–0.15) | 0.99  (0.98–1.00) |

pos: positive. neg: negative. PPV: positive predictive value. NPV: negative predictive value.

Data are presented as n or probability with 95% confidence interval.
